# Exploring the Applications of the Photoprotective Properties of Anthocyanins in Biological Systems

**DOI:** 10.3390/ijms21207464

**Published:** 2020-10-10

**Authors:** Hélder Oliveira, Patrícia Correia, Ana Rita Pereira, Paula Araújo, Nuno Mateus, Victor de Freitas, Joana Oliveira, Iva Fernandes

**Affiliations:** LAQV, REQUIMTE, Departamento de Química e Bioquímica, Faculdade de Ciências, Universidade do Porto, Rua do Campo Alegre s/n, 4169-007 Porto, Portugal; helder.oliveira@fc.up.pt (H.O.); patricia.correia@fc.up.pt (P.C.); anarita@fc.up.pt (A.R.P.); paula.araujo@fc.up.pt (P.A.); nbmateus@fc.up.pt (N.M.); vfreitas@fc.up.pt (V.d.F.)

**Keywords:** anthocyanins, photoprotection, skin aging, eye diseases, photodynamic therapy

## Abstract

Due to their physical and chemical characteristics, anthocyanins are amongst the most versatile groups of natural compounds. Such unique signature makes these compounds a focus in several different areas of research. Anthocyanins have well been reported as bioactive compounds in a myriad of health disorders such as cardiovascular diseases, cancer, and obesity, among others, due to their anti-inflammatory, antioxidant, anti-diabetic, anti-bacterial, and anti-proliferative capacities. Such a vast number of action mechanisms may be also due to the number of structurally different anthocyanins plus their related derivatives. In this review, we highlight the recent advances on the potential use of anthocyanins in biological systems with particular focus on their photoprotective properties. Topics such as skin aging and eye degenerative diseases, highly influenced by light, and the action of anthocyanins against such damages will be discussed. Photodynamic Therapy and the potential role of anthocyanins as novel photosensitizers will be also a central theme of this review.

## 1. Introduction

**Figure 1:**
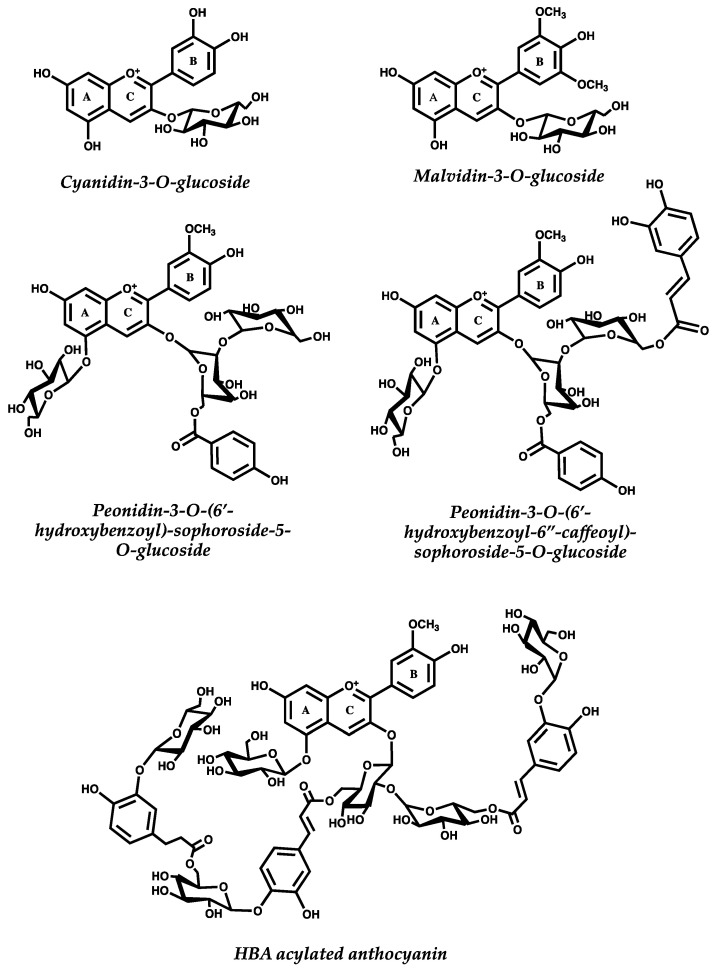
Anthocyanins structure from different sources. Cyanidin-3-O-glucoside is the most abundant anthocyanin in nature and can be found in several fruits. Malvidin-3-O-glucoside is the main anthocyanin from Red Wine and grapes from Vitis vinifera [4]. Peonidin-3-O-(6’-hydroxybenzoyl)-sophoroside-5-O-glucoside and Peonidin-3-O-(6’-hydroxybenzoyl-6’’-caffeoyl)-sophoroside-5-O-glucoside are normally found in vegetables such as Purple Sweet Potato [5]. HBA acylated anthocyanin—a 3-O-(2-O-(6-O-(trans-3-O-(β-D-glucopyranosyl)-caffeoyl)-β-D-glucopyranosyl)-6-O-(trans-4-O-(6-O-(trans-3-O-(β-D-glucopyranosyl)-caffeoyl)-β-D-glucopyranosyl)-caffeoyl)-β-D-glucopyranosyl)-5-O-(β-D-glucopyranosyl)-Peonidin—is typically found in Heavenly Blue Morning Glory Ipomoea tricolor flowers [6].

Anthocyanins are one of the most intriguing class of polyphenols. These compounds are usually referred as one of the largest groups of water-soluble natural phenolic compounds, which come from their glycosylated form [1]. Anthocyanins are present in a wide variety of plants and fruits, being responsible for their colors, ranging from red to blue. Their structure is based on the core skeleton of flavonoids [2] and thus, chemically, anthocyanins are 2-phenylbenzopyryliums, consisting of an aromatic ring (A) bonded to a heterocyclic pyran (C) that is in turn bonded to an aromatic ring (B) bonded by a carbon–carbon connection. The aromatic ring B can have different hydroxylation and methoxylation patterns, while hydroxyl groups present at C3, C5, and C7 carbons of the core structure can be glycosylated by different sugars [3]. Only the glycosylated structures are known as anthocyanins, while the non-glycosylated are the anthocyanidins and considered precursors of anthocyanins. From simple monoglucosides to complex structures involving multiple glycosylation and acylation patterns, anthocyanins have a wide variety of structural features, and up to date more than 700 have been described [3]. They are derived from six common anthocyanidins present in nature: pelargonidin, cyanidin, delphinidin, peonidin, petunidin, and malvidin. Depending on the source, these glycosylated products of anthocyanidins present different substitution patterns. In fruits such as berries or grapes, their structures are usually monoglucosides that can be esterified with different acids. In vegetables and plants, anthocyanins tend to have structures with a high molecular weight involving multiple glycosylation sites and glycosyl acylations. Figure 1 shows anthocyanins from different sources based on their structural complexity.

Anthocyanins have been reported as potential health bioactives, revealing several biological properties. They present anti-inflammatory effects, modulation of tumor growth, anti-diabetic properties, and also cardiovascular and neurological protective activities [3,7,8,9]. They have also been reported as antimicrobial modulators [10]. Such properties are usually attributed to their antioxidant activity [3,11]. Due to such reasons, the exploitation of other biological properties has been the focus of several research works over the last few years. Good examples of this, are the anti-aging actions of anthocyanins in skin and their health benefits to the eye. Anthocyanins have presented UV photoprotective effects in human dermal fibro and protection effect against light-induced damage in human retinal cells [12,13,14,15]. Anthocyanins are the only class of polyphenols able to absorb light in the UV (280–400 nm) and blue light region (360–500 nm) [16]. Altogether these properties may account for anthocyanins and potential on photoprotection.

Therefore, in this review, we highlight the photo and chemical properties of these compounds, and the recent findings and perspectives on the potential use of anthocyanins for photoprotective applications either in preventive or therapeutic approaches.

## 2. Chemical Equilibria and Stability of Anthocyanins

**Figure 2:**
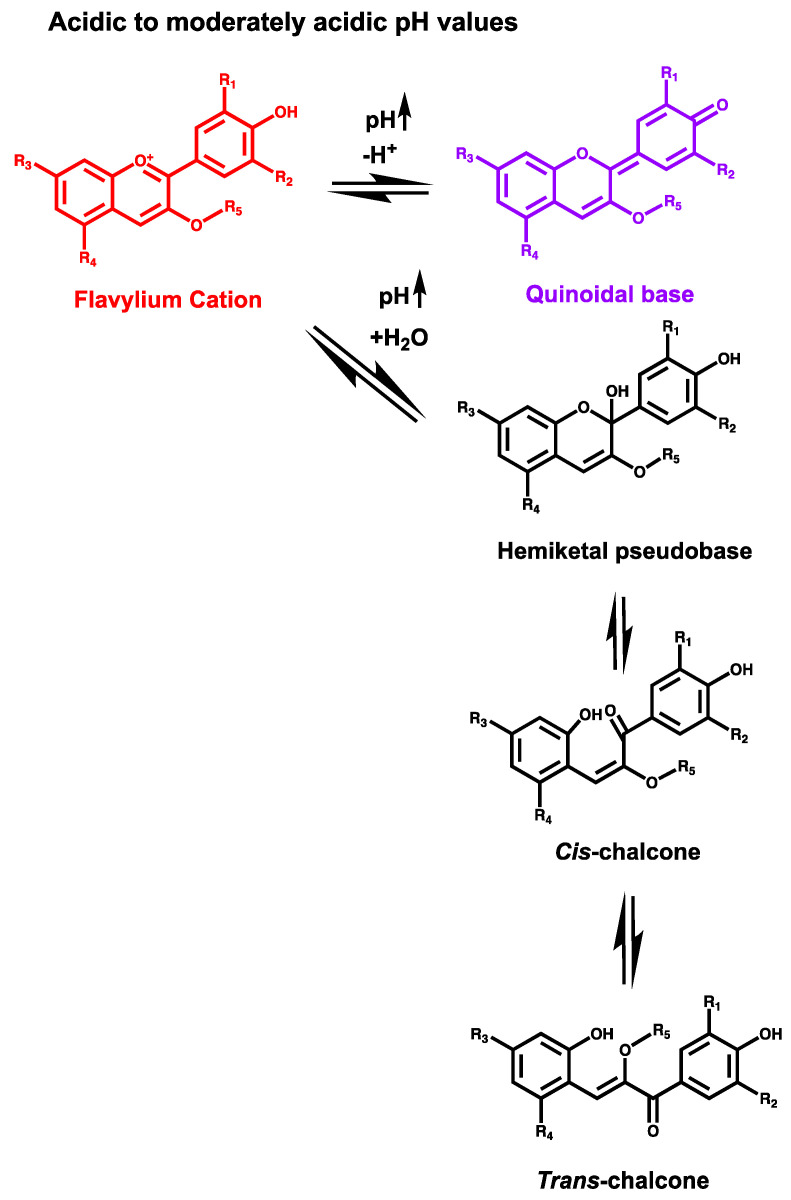
Dynamic equilibrium network of anthocyanins at different pH values. R_1_ = H, OH or OCH_3_; R_2_ = H, OH or OCH_3_; R_3_ = OH or sugar moiety; R_4_ = OH or sugar moiety; R_5_ = sugar moiety. The sugar moiety of anthocyanins can be composed by different attached molecules.

Anthocyanin’s structure is pH-dependent, meaning that they can adapt to different environments, translating not only in different forms but also in different visible colors [6,17]. Such a fact attracted the scientific community to study how these phenomena happen, and nowadays the chemical pH dependence of anthocyanin’s structure is well understood [18,19,20,21,22,23]. They are generally represented by their flavylium cation form (Figure 1). However, this is only predominant at very acidic pH values. Anthocyanins rather exist in an equilibrium network between different structural forms depending on the pH environment (Figure 2).

At pH 1, anthocyanins are in their most stable form, the flavylium cation. With the increase of pH values, the structure of anthocyanins is delocalized to a proton-transfer where the flavylium cation donates a proton to give origin to a neutral quinoidal base with a bluish color. Concomitantly, a slower hydration process occurs in which a hydroxyl group from water is delivered to the cationic structure to originate a neutral hemiketal pseudo-base. This structure establishes a ring-opening equilibrium in a period of sub-seconds, tautomerizing to originate a *cis*-chalcone structure. After this and in a much slower pace, the *cis*-chalcone will eventually isomerize to originate *trans*-chalcone. This is of particular importance to understand the biological properties reported for these compounds, once in vivo, anthocyanins are subjected to different pH environments. It is also important to notice that the exact pH at which one structure or the other will be predominant will highly depend on the substitution patterns of the flavanic core, which will influence the kinetic and thermodynamics parameters [17].

The stability of these compounds is also influenced by other factors rather than pH, namely temperature, concentration, light, oxygen, solvents, presence of enzymes, metals or other ions [24].

Recently, anthocyanins from blueberries, were found to degrade rapidly to 25% of the initial amount at 60 °C [25], while the half-time degradation of anthocyanins from Purple Sweet Potato was reported to be 111.6 h at the same temperature [26]. In blueberries, the anthocyanins are mainly monoglucosides with different sugar moieties, while in purple sweet potato, anthocyanins are usually glycosyl-acylated derived from peonidin and cyanidin with multiple sugar moieties, thus with a much higher degree of complexity. Furthermore, in fact, this type of anthocyanin was reported to have an overall enhanced resistance [27]. Light is also an important factor for their stability. While it is important for the biosynthesis of these compounds in plants, it can also accelerate their degradation in vitro, in biological tissues, and in different foodstuffs [28,29]. In addition, oxygen deleterious action can be as big as the influence of pH on anthocyanins and occurs either by direct action or by the formation of radicals [30]. There are also strategies to enhance anthocyanins chromatic stability such as the formation of aggregates at higher concentrations and their complexation with non-colored compounds such as caffeine [31], hydroxycinnamic acids, flavonols, and some metal ions [32,33]. Both of these strategies appear to potentiate the color yield of these compounds in solution, prompting a higher stability of their quinoidal structures.

For anthocyanin derivatives, the role of these factors can also represent an important parameter for their overall stability. However, due to their structural features, they tend be more stable than the parent compounds when subjected to the same conditions [34,35,36].

## 3. Photochemical Properties of Anthocyanins

**Figure 3:**
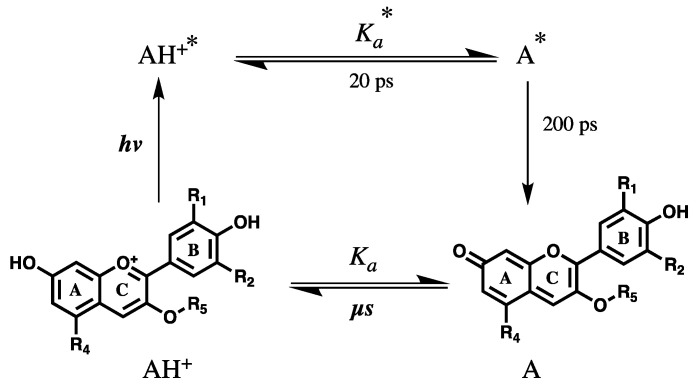
Ground- and excited-state proton transfer process of anthocyanins. Absorption of light (hν) by the ground state cation form (AH^+^) produces the first excited singlet state of the cation (AH^+^*)**,** which transfers a proton to water to form the excited singlet state of the conjugate base A*. The excited base form (A*) lives about 200 ps before transforming the excitation energy into heat and returning to the ground state of the base (A), which then reprotonates back to AH^+^ with no net chemistry. R_1_ = H, OH or OCH_3_; R_2_ = H, OH or OCH_3_; R_4_ = OH or sugar moiety; R_5_ = sugar moiety. The sugar moiety of anthocyanins can be composed by different attached molecules. The ESPT (acid dissociation constant in the excited state, Ka*) occurs on the picosecond time-scale and ground-state proton transfer (acid dissociation constant in the ground state, Ka) on the micro- to nanosecond time-scale.

The impact of light on the multistate equilibrium of anthocyanins has been reported by several authors. Any photo-chemical study concerning anthocyanins, in aqueous solution, must consider their extremely rich and interesting ground- and excited-state chemistry [19,20,22,37,38,39]. In the ground state, as discussed before, natural anthocyanins can exist in acidic aqueous solution (pH < 7) in at least five different forms that are coupled to each other via pH-dependent equilibria [38,40,41], while in basic solution this number can increase due to additional deprotonation equilibria [42]. Besides this, as previously mentioned, anthocyanins in nature are present as mixtures of the different basic structures in different degrees and types of glycosylation, and in the presence or absence of additional acyl groups attached to the sugars, which represents a barrier to systematic studies of the chemistry and photochemistry of natural anthocyanins. Chemistry and photochemistry features of naturally-occurring anthocyanins are usually mimicked by using synthetic 7-hydroxyflavylium cations [39], which correspond to the basic chromophore of anthocyanins. Upon absorption of light in aqueous solution, anthocyanins and 7-hydroxyflavylium cations in the first excited singlet state behave as superphotoacids (pKa* < 0) [43] and the lifetimes of the excited acid form (AH^+^*) are extremely short, in about 5–20 ps [44,45,46,47,48] due to ultrafast adiabatic proton transfer to water to form the corresponding excited base (A*) [49] (Figure 3). The excited-state lifetimes of the conjugate bases (A*) are also quite short, in the order of 200 ps, resulting in rapid decay to its ground state [44,45,46,47,48]. Thus, excited-state proton transfer (ESPT) serves as a very efficient pathway for the ultra-rapid conversion of the absorbed light energy into heat, with subsequent return to ground state, (A), which then reprotonates to (AH^+^), at a rate dependent on the pH of the solution. Such a process may be at the origin of anthocyanin’s photoprotective role in plant tissues when submitted to excessive solar radiation.

In the ground state, anthocyanins can also form complexes with colorless electron-rich “copigments” molecules such as hydroxybenzoic or hydroxycinnamic acids and others; and on the other hand, acyl anthocyanins with one or more copigment molecules covalently attached to the sugar residues can result in intramolecular copigmentation complexes. The charge–transfer interactions involved in these copigmentation complexes increase the stability of the flavylium cation form, which increases the value of the pK_h_ and opens up a new charge–transfer mediated channel for direct conversion of the absorbed light energy into heat that is even faster (1 ps) than excited state proton transfer [50]. As previously reported, the isomerization process of anthocyanins and other flavylium compounds can occur thermally or photochemically. The irradiation of *trans*-chalcone yields to the formation of a *cis* isomer, which can back again to *trans*-chalcone or originate flavylium cation/quinoidal base via tautomerization and dehydration. Moreover, flavylium compounds are interesting examples of multistate/multifunctional systems that, usually, combining two different stimuli—pH and light, can be used in information processing, like in optical memories models capable of write-read-erase [51]. The presence of a thermal barrier between *cis*-*trans* isomers is one of the necessary conditions of these system, because otherwise the signal auto-erases. However, it is not easy to achieve that since the photochemical reaction leads to the formation of a metastable state, which tends to revert back to the initial and thermodynamically stable *trans* isomer [39]. Anthocyanins exhibit a high *cis*-*trans* isomerization barrier and are poor photochromic systems due to the presence of a negligible amount of *trans*-chalcone at equilibrium. The best photochromic systems that have been described for synthetic flavylium are 4′-hydroxyflavylium, 4′-methoxyflavylium, 4′,7-dihydroxyflavylium, and others [52].

## 4. Photo-Oxidative Damage and the Role of Anthocyanins

### 4.1. Skin Aging

Skin aging is a complex biological process that leads to progressive deterioration of cutaneous structures and functions over time [53]. Intrinsic skin aging is determined by an individual’s physiological predisposition and develops from the combination of genetic factors and hormonal and metabolic variations, that typically manifest in the form of fine wrinkling and thinning of the skin [54,55]. Exposure to extrinsic harmful factors is also a major contributor to the alterations on skin structure and function. Chronical sun exposure, the main source of ultraviolet radiation (UVR), is the predominant cause of oxidative stress in the skin and one of the most extensively studied factors contributing to the aging process.

The more prominent structural changes occur within the dermis, the layer that gives the skin its strength, elasticity, and firmness, mainly consisting of an extracellular matrix (ECM) made of collagen and elastin and to a lesser extent, proteoglycans and glycosaminoglycans, which provide hydration to the skin due to their great water retention capacity [56]. Matrix metalloproteinases (MMPs), a family of ubiquitous endopeptidases, play a vital role in the molecular mechanisms underlying ECM protein degradation as their expression has shown to be considerably elevated during the aging process, particularly in photodamaged skin [57]. Reactive oxygen species (ROS) constitute the major driving force behind the increase of MMP levels [58].

Briefly, UVR incidence induces ROS production within keratinocytes and dermal fibroblasts, promoting the activation of specific signaling pathways which results in upregulated expression of two key transcription factors: activator protein 1 (AP-1) and nuclear factor-κB (NF-κB). Increased activity of both enhances the expression of several MMPs involved in collagen degradation, including MMP-1 (interstitial collagenase), MMP- 3 (stromelysin), and MMP-9 (gelatinase), resulting in accumulation of fragmented and irregularly distributed collagen fibrils and compromising the structural integrity of the dermal ECM [56,57,59,60]. AP-1 has also been demonstrated to reduce the production of procollagens by inhibiting transcription of genes encoding procollagen I and III in fibroblasts [61]. Simultaneous increased collagen degradation and reduced synthesis hinders the mechanical interaction between fibroblasts and the ECM, leading to a reduction in the size and elongation of fibroblasts and collapsed morphology. Aged fibroblasts produce a greater amount of ROS that further stimulate the above-mentioned molecular mechanisms, creating a feedback loop that accelerates the aging process [56,62]. The elastic fiber system also suffers significant structural changes as a result of MMPs upregulated expression. MMP-12 (macrophage elastase) plays a crucial role in elastin degradation and development of solar elastosis, a hallmark of photoaging, that consists of an abnormal accumulation of coarsen, disorganized and nonfunctional elastic fibers [59,63]. Overall, these mechanisms typically emerge in the form of deep wrinkling, laxity, severe atrophy, and leathery appearance [64].

Skin is endowed with efficient antioxidant molecules with the ability to respond to UVR-triggered generation of ROS, such as superoxide dismutase (SOD), catalase (CAT), and glutathione peroxidase (GSH-Px) [65]. However, when the abnormal increase of ROS overwhelms this defense capacity, the skin is unable to prevent oxidative damage of cellular membranes, genomic DNA (which can eventually cause mutations on oncogenes and tumor suppressor genes that prompt inappropriate survival and proliferation of skin cells, a step towards initiation and progression of skin carcinogenesis), intracellular proteins, and lipids [66,67]. The network of antioxidants of the skin is damaged as well, accentuating the disparity between the production and elimination of ROS. Moreover, UVR stimulates the production and secretion of certain proinflammatory mediators in fibroblasts and particularly in keratinocytes, including prostaglandin E2 (PGE2), tumor necrosis factor (TNF-α), and interleukin-1 (IL-1). These mediators consequently trigger molecular pathways that result in decreased synthesis of collagen and increased MMPs activity and they can further amplify the inflammatory response by binding to their specific receptors on neighboring skin cells or on the cells where they were originally produced, intensifying the skin damages [58,68,69,70].

Another relevant feature and cosmetic concern associated with photoaging is the manifestation of skin hyperpigmentation. Melanin is synthesized in epidermal melanocytes, within specialized organelles termed melanosomes, and subsequently transferred to neighboring keratinocytes where it accumulates and shields the nuclear DNA by absorbing and scattering UVR [71,72]. A complex network of keratinocyte and fibroblast-secreted factors such as endothelin-1, alpha stimulating hormone (α- MSH), and stem cell factor (SCF) are described as modulators of the function of melanocytes [73,74]. Increased synthesis of these factors upon continuous exposure to UVR is associated with increased melanogenesis, melanocyte density, and transfer of melanosomes to keratinocytes, resulting in pigmentation disorders such as melasma and solar lentigines [75,76].

This is the mechanistic basis that explains the boosting capacity of UVR in skin aging and the reason why people, even at a relatively young age, often exhibit noticeable skin damages.

Natural bioactives have been drawing attention do to their photoprotective and antiaging capacities, including anthocyanins. Due to their previously stated physical-chemical characteristics, these compounds constitute a potential source of anti-aging modulators. In fact, several studies mostly using cellular and animal skin models have revealed promising and elucidating results about the pharmacological mechanisms by which anthocyanins prevent UV-induced skin damage [77].

In a study conducted by F Afaq et al. pretreatment of HaCaT human keratinocytes with delphinidin reduced the extent of UVB-induced formation of apoptotic cells from 25.72% to 7.69%. Similarly, microscopical analysis of SKH-1 hairless mouse skin biopsies revealed that topical application of delphinidin inhibited the formation of apoptotic cells and the formation of 8-hydroxydeoxyguanosine (8-OHdG), an important biochemical marker of UVR-induced oxidative damage in DNA [78]. Furthermore, delphinidin prevented UVB-induced MMP-1 expression evidencing its capacity to counteract the collagen breakdown and consequent formation of wrinkles. It is suggested that delphinidin directly inhibits an endogenous generator of ROS, NADPH oxidase, whose activity is known to be increased by UVR skin exposure and is associated with MMP-1 upregulated gene expression [79]. In another study, the same anthocyanidin was found to inhibit UVB-induced cyclooxygenase-2 (COX-2) expression and a consequent production of its primary product, PGE2, both in murine epidermal cells and mouse skin [80]. Cyanidin has been demonstrated to suppress the same inflammatory mechanism, both in murine and human keratinocytes [81,82].

As mentioned earlier, overproduction of PGE2 is known to significantly damage and age skin and also plays a well-recognized role in the development of premalignant lesions and skin cancer [83].

An innovative system based on a topical formulation containing deformable liposomes loaded with blueberry anthocyanins (nanoberries) was proposed as a strategy to enhance the penetration of the *stratum corneum* and provide in situ skin photoprotection. Nanoberries were successfully incorporated into HaCaT keratinocytes and protected the cells from UVR exposure, while exhibiting minimal cytotoxicity [84]. This concept could also provide a more efficient delivery of anthocyanins into the deeper layers of the skin. Although UVB is reported to be particularly damaging to the epidermis, it is also known as a key contributor to dermis photoaging by activating MMPs and blueberry anthocyanins have been demonstrated to inhibit UVB-induced damage in human fibroblasts, meaning this nano-strategy could possibly enhance their dermis protective capacity [85].

A very recent study reported the in vivo protective effects of anthocyanin extracts from purple fleshed sweet potato (PSP-AE) in a murine model after UVB irradiation for eight weeks. PSP-AE treatment effectively reduced the UVB-induced epidermal hyperplasia, improved the skin hydration, and inhibited collagen fibers network degradation. Interestingly, it was shown that the attenuated MMP-1 protein expression was concomitant with the inhibition of MAPK and NF-κB signaling pathways [86]. Purple sweet potato anthocyanins have particular properties regarding pH and heat resistance, photosensitivity, and overall stability due to the acylation with phenolic acids such as caffeic acid, *p*-coumaric acid, and ferulic acid, which could be advantageous to preserve their natural colors and desired effects in a broad range of applications, including the cosmeceutical industry [87]. Besides, it has been reported that the presence of these aromatic organic acids in the structure of anthocyanins enhances their natural UV absorption capacity, thus representing a more efficient mean of photoprotection [88,89]. In fact, there has been a growing interest in exploring the use of anthocyanins in UV-blocking formulations, especially due to raising awareness of the safety and toxicity issues associated with the synthetic compounds conventionally used in sunscreens.

Chan et al. demonstrated in a preliminary in vitro chemical study that the addition of anthocyanin extracts of purple sweet potato to a cosmetic formulation improved its UV absorption ability, indicating the potential topical use of anthocyanins as natural sunscreens [90]. In another study, blackberry and raspberry extracts of anthocyanins were tested for the same purpose and the in vitro solar protection factor (SPF) was found to be 54.57 for blackberry and 37.32 for raspberry, promising values for new sun filters development. Overall, the formulations containing the extracts exhibited pH and density stability, pink color, and creamy aspect, although indirect light and stove conditions resulted in a slight color change, suggesting they should be kept under refrigeration and in opaque package to ensure stability [91]. Additionally, the photoprotective effects of strawberry-based formulations enriched with Coenzyme Q10 (CoQ10) were tested in UV exposed human dermal fibroblasts. Cells treated with the formulations with higher concentrations of strawberry extract, containing pelargonidin and cyanidin glycosides as the most representative anthocyanin components, were able to protect the cells from UV radiation harmful effects, restoring the cellular viability to similar values of those observed in non-irradiated cells, demonstrating that the topical use of strawberry extract may provide good photoprotection [92].

Furthermore, the application of anthocyanins has also been explored as a skin whitening agent to reduce hyperpigmentation, a common evidence of photoaged skin. Tyrosinase is a major rate-limiting enzyme of melanin biosynthesis, therefore tyrosinase inhibitors have been extensively explored for the treatment of dermatological issues, such as solar lentigines and melasma. Multiple studies have reported the anti-tyrosinase in vitro activity of anthocyanins [93,94,95]. A recent research using anthocyanins from the *Hibiscus syriacus* L., the Korean national flower, evidenced their capacity to decrease melanin production both in α-MSH stimulated B16F10 murine melanocytes and zebrafish larvae. Curiously, the mechanism behind inhibition of melanogenesis was described as being the anthocyanin-induced phosphorylation and consequent degradation of Microphthalmia-Associated Transcription Factor (MITF), a cell-specific factor responsible for the transcriptional activation of tyrosinase gene and not through direct inhibition of tyrosinase activity [96,97].

Taken together, these results strongly support the role of anthocyanins as photoprotective agents, either by absorbing UV radiation or by attenuating events exacerbated by cumulative UVR exposure.

### 4.2. Eye Degenerative Disease

#### 4.2.1. Light-Induced Damage to the Retina

Retinal degenerative diseases are multifactorial diseases with a complex pathophysiology, in which excessive production of ROS and oxidative stress play a crucial role in the onset and progression of this disease [98]. Crosstalk between oxidative stress-related and inflammatory pathways is likely to be important in inducing blood retinal barrier (BRB) breakdown and pathological neovascularization. A better understanding of this dysfunction and the elucidation of the complex crosstalk between nutrition and the initial stages of disease progression may facilitate the development of novel personalized diets with significant clinical benefit in comparison with existing therapies. Simultaneous, chronic interventions addressing multiple metabolic and signaling pathways may be beneficial to attenuate oxidative stress and inflammation and ameliorate the retinal injury. Considerable research has focused on the role of diet and light exposure. Retinal cells are susceptible to photooxidative damage, during which high amounts of reactive oxygen species are generated and these oxidative damages contribute to the progression of retinal cell degeneration [99].

Photochemical damage is the most common form of retinal damage caused by exposure to direct sunlight and several artificial light sources. Photochemical damage occurs when light is absorbed by a chromophore and leads to the formation of an electronically excited state of that molecule, which then undergoes either chemical transformation itself and/or interacts with other molecules leading to chemical changes of both interacting molecules or to a transfer of the excitation energy to the other molecules.

The retina contains a number of endogenous photosensitizers which can be excited by visible/infrared light reaching the retina. The outer retina (photoreceptors and retinal pigment epithelium (RPE)), is immediately adjacent to the choroidal blood supply and thus highly oxygenated. Therefore, these are potentially favorable conditions for photodynamic damage to occur at the photoreceptors at irradiations with short wavelength light (320–440 nm) and to the pigment epithelium at longer wavelengths, the blue light damage (>440 nm) [100].

Furthermore, this type of damage appears to be oxygen-dependent, since elevated blood oxygen has been reported to increase retinal photosensitivity [100]. Protective effects of antioxidants and lowering oxygen tension suggest that this type of light damage is due to photodynamic damage in the retina [100].

#### 4.2.2. Endogenous Photosensitizers

Not all retinal cells are typically susceptible to damage from light. Inner retinal cells such as ganglion cells, Müller cells, amacrine cells, and bipolar cells, are not known to be directly involved in phototoxicity. Instead, rods and cones, which require photopigments to absorb photons as the first step in seeing, are much more likely to be damaged by excess amounts of visible light. Similarly, the RPE cells contain light absorbers such as melanin, lipofuscin, and retinoids, which make them susceptible to photochemical damage. Although the exact mechanism is still unknow, it is believed that there are two possible mechanisms for photochemical retinal damage [101]. Class I damage has an action spectrum that suggests the involvement of photoreceptor photopigments. Class II or “blue-light” damage, has a damage threshold action spectrum that increases with wavelength and may be linked to chemical changes in lipofuscin.

All visual pigments in vertebrates are formed by a transmembrane protein, opsin, which binds via a Schiff base linkage of its lysine residue to 11-cis-retinal. The visual pigment of rods is named for its color rhodopsin. There are three types of cones in human retina, the blue cone (419 nm), the green cone (531 nm), and the red cone (558 nm).

The 11-cis-retinal, while attached to opsin in rhodopsin is isomerized to all-trans-retinal by light. Free all-trans-retinal is not only toxic as a reactive aldehyde and, in the presence of redox active metal ions, a source of free radicals in dark, but it is also a potent photosensitizer photoactivated by UV-A and blue light [101]. Photoexcitation of all-trans-retinal with UV-A or blue light is followed by an efficient intersystem crossing from an excited singlet state and formation of an excited triplet state. The energy of retinal triplet state is high enough to enable an efficient energy transfer to molecular oxygen and, as a result, a singlet oxygen is produced. Unless effective antioxidants and repair enzymes counteract it, ROS induce oxidative damage to lipids and proteins, which may affect their structures and functions.

Lipofuscin accumulates with age in the lysosomes of the RPE as a by-product of the visual cycle and phagocytosis [101]. These autofluorescent yellow-brown pigments granules appear to be the product of the oxidation of unsaturated fatty acids and may be symptomatic of membrane damage, or damage to mitochondria and lysosomes. Lipofuscin is highly susceptible to photochemical changes that may lead to irreparable cellular damage and may be the photosensitizer for class II.

#### 4.2.3. Anthocyanins as Retinal Chromophores

Anthocyanins are the only class of polyphenols able to absorb light in the UV (280–400 nm) and blue light region (360–500 nm) [16], which may account for anthocyanin’s protection effect against light-induced damage in human retinal cells [12,13,14]. In fact, the benefits on vision and eye health of anthocyanins was one of the 1st reported properties of anthocyanins that kept attracting the interest of consumers and the scientific community [102]. More attention has been paid to anthocyanins effect on vision after the hypothesis of improved night vision of British Royal Air Force aviators in World War II in association with their regular ingestion of bilberry jam at breakfast [102].

Up till then, several in vitro and in vivo studies have reported antioxidant and protective effects at the eye level for anthocyanins. For example, anthocyanins from blackcurrant and blueberry extracts have demonstrated antioxidant effects at the level of RPE cells by inhibiting the photooxidation of the A2E molecule and neutralizing oxygen free radicals [103,104]. In vitro studies on Maqui berry extract have shown that it can protect the retinal cells against light-induced photoreceptor degeneration due to its content of anthocyanins [105]. Malvidin and its glycosides increase SOD and catalase in high glucose-induced human retinal capillary endothelial cells, protecting these cells from oxidative stress-induced damage, and by anti-inflammatory properties due to inhibition of ICAM-1 and NF-κβ [106].

Other studies performed on rats have shown that anthocyanins from blueberry extract can inhibit diabetes-induced retinal abnormalities [107,108]. Moreover, anthocyanins extracted from black soybean seeds have shown their protective effects on rat retinal neurons damage induced by N-methyl-N-nitrosourea [109].

Besides all the proposed mechanisms for in vitro protective effects of anthocyanins on eye health, some clinical studies focused on the use of anthocyanins to improve night vision have retrieved negative outcomes [110]. However, the role of anthocyanins in vision health is still controversial. The conflicting conclusions and results are due to the disparity between in vitro and in vivo studies, generally limited to acute studies relevant only to pharmaceutical research. Further studies will be needed to establish the pleiotropic mechanisms of anthocyanins and to show how they can practically interfere in different visual processes [111].

Furthermore, there is a mis-consideration of anthocyanins unique physical-chemical properties that clearly affect their behavior in vivo. As already referred to, anthocyanins may occur under different equilibrium structures that may have a different biological influence depending on the surrounding pH [16]. Moreover, they are also very unstable at neutral pH and physiological temperature and can interact with proteins or carbohydrates [112]. In addition, the bioavailable forms of anthocyanins in vivo are not exclusively the same that occur in food, since they are also largely metabolized yielding several types of metabolites [113]. These include anthocyanins phase II metabolites (glucuronide, methylated, and sulfate) or simpler phenolics, that include phenylpropionic, phenylacetic, benzoic acids, and m-hydroxyphenylpropionic acids, intact or their phase II metabolites, which can be absorbed by enterohepatic recirculation [114,115,116]. Total concentration of anthocyanins in several ocular tissues is higher than that measured in plasma, a finding that suggests that anthocyanins can concentrate in ocular tissues [117]. In an experimental study by Jang et al., the accumulation of anthocyanins in the eye tissues of animals (after 4 weeks of dietary supplementation with blueberries) exhibited ocular protective effects and reversal of oxidative effects [118]. Significantly high concentrations of anthocyanins in some ocular tissues are measured when anthocyanins are administered either intravenously or intraperitoneally [117], which reinforces the importance of metabolites and catabolites in ocular disease prevention. Systemically, metabolites have limited diffusion into the eye due to the presence of various ocular barriers. The retinal endothelium acts as a selective barrier (inner Blood Retinal Barrier) between the blood circulation and the neural retina. The intact forms of anthocyanins, with a sugar moiety are not likely to cross cell barriers by diffusion. However, and similar to plasma glucose, anthocyanins may be transported across the cell membrane through facilitative glucose transporters, namely the constitutive isoform GLUT1 or other isoforms, which may provide a route for a higher bioavailability of anthocyanins phase II metabolites in the BRB [119]. Glucuronidation is the major route for metabolization of anthocyanins after ingestion in human intervention studies [120]; these metabolites are also able to be transported by GLUTs [121]. Table 1 shows a summary of the photoprotective effects of anthocyanins in different models.

## 5. Photodynamic Therapy

### 5.1. Photodynamic Therapy Mechanism

**Figure 4:**
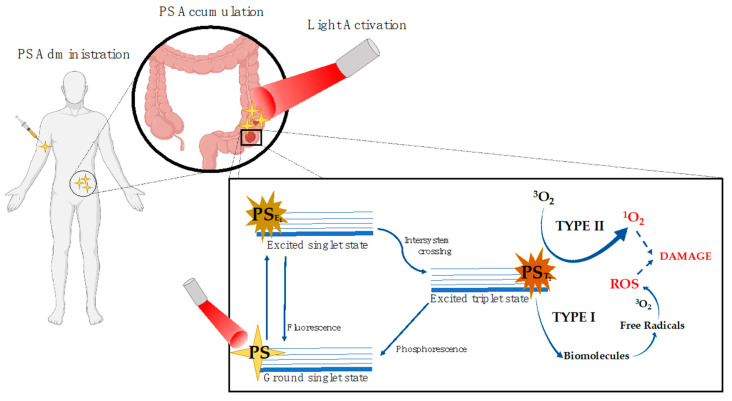
Photodynamic Therapy mechanisms. The photosensitizer (PS) is administered and distributed systemically. After, it starts to accumulate in the target damaged tissues. The PS is finally activated by the appropriate absorption wavelength light and the triplet state PS can go either type I or type II mechanism.

Photodynamic therapy (PDT) was discovered more than 100 years ago and, over the past few years, it has been thoroughly investigated and developed gaining a status of a consolidated technology for the treatment of different pathologies. The principle behind PDT is based on the photo-activation of certain compounds (photosensitizers) after their local or systemic application and optimum accumulation on pathological tissues. The process involves the activation of the compounds through the absorption of light with specific wavelengths and the initiation of a series of biochemical processes leading to the destruction of the damaged tissue. Such activation can occur by two distinct mechanisms dependent on the intracellular oxygen pressure. For that, the photosensitizer is irradiated, after entering the cells, with the corresponding light absorption wavelength. This phenomenon allows the excitation of the photosensitizer and upon the radiation step, part of the energy is radiated in the form of a quantum of fluorescence while the remaining directs the photosensitizer to the triplet state, which represents the bioactive and therapeutic form of the molecule [122]. In type I, a radical mechanism through the electron/hydrogen transfer occurs by direct-contact reactions (between the PS and the tissue) (Figure 4), leading to the initiation of radical chain reactions and direct damage to the biomolecules. This happens due to the triplet state of the photosensitizer that is able to transfer energy to the biomolecules. The electronic/protonic transfer will create free radicals and PS and substrate anions then can react with the oxygen molecules present within the damaged tissue, yielding to the formation of ROS that will further develop intracellular ROS cascades culminating in oxidative stress and the destruction of the damaged cells. The type II mechanism involves the transfer of the excitation energy directly to the triplet oxygen molecules originating singlet oxygen (Figure 4). This specie is highly reactive due to its electrophilic behavior being capable of damaging different cellular components such as proteins, membranes, or even genetic material. This direct energy transfer is possible if the PS has the same spin of the molecular oxygen [123,124]. Both of them culminate in the production of ROS, and given the short lifetime of these species, PDT can produce a strong localized effect [125].

**Figure 5:**
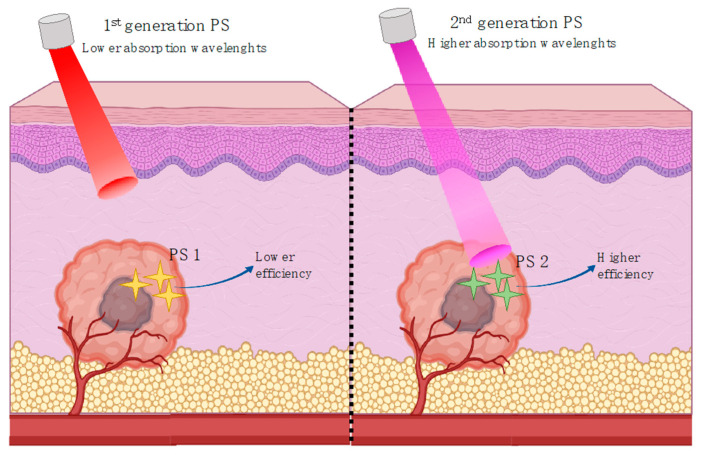
Penetration capacity of different absorption wavelength lights for 1st generation PS and 2nd generation PS. At higher wavelengths, the light can have a deeper penetration through tissues therefore enhancing the activation of the photosensitizer.

The prevalence of either mechanism will depend on several factors, such as oxygen concentration, tissue dielectric constant, and pH and photosensitizer structure. While the direct-contact reactions resulting from type I mechanisms usually cause higher severity damage, they also cause photodegradation of the PS. On the other hand, the direct transfer between the PS and oxygen molecules promotes the replenishing of the PS. Nevertheless, it is clear that the role of the PS is efficient for the success of PDT. PS molecules must be able to accumulate preferentially in damaged cells, such as in cancer. Furthermore, in fact, the common PS tend to combine preferentially with low density lipoproteins, highly increased in damaged tissues [126,127]. This tendency makes membranes of particular interest to PDT efficiency. The oxidations promoted on lipids through the action of the photosensitizers promotes membrane disruption, caused by the presence of free radicals and oxygen singlets [128]. Consequently, PSs with a higher degree of accumulation in the damaged cells may potentially exhibit greater cytotoxicity such as demonstrated for cationic porphyrins in Hep2 cells [129]. The research for new PS with a higher efficiency, in the hope to overcome the inherent problems of PDT has establish three generations for these compounds. The first-generation compounds englobe the first attempts of PDT clinical approaches. Hematoporphyrin was the first compound used as a PS. Furthermore, some of the most still commonly used compounds such as Photofrin are first-generation [130]. However, the first-generation compounds generally present low chemical purity and high half-life times, needing a higher time of light incidence, and consequently causing problems such as hypersensitivity in skin. Furthermore, the poor penetration of light represents a problem at the maximum absorption wavelengths of these compounds (Figure 5). For the second-generation of compounds research initiated with a need to overcome such problems and compounds with higher purity, higher yield of singlet oxygen and better maximum absorption wavelengths for a deeper penetration capacity (in the range of 650–800 nm) has been reported [131,132,133,134,135,136,137,138,139]. Such compounds have also an overall poor aqueous solubility making the search for new methods of delivery necessary. From the second-generation compounds, 5-aminolevulinic acid (ALA) became an important example. Its metabolization allows this compound to become an active PS only after being transformed in a protoporphyrin and has been used in different clinical applications [140,141]. The third generation has been focusing on the discovery of compounds with higher affinity to the target damaged tissue, thereby reducing the damage to the surrounding healthy cells. This includes not only the synthesis of new molecules but also the combination of pre-existing compounds (first and second-generation) with drug delivery systems to improve their bioavailability, therefore improving the efficacy of PDT. The most outstanding outcomes of these efforts may be the Lab-on-a-chip systems utilizing different nanomaterials in combination with several drugs to improve the efficacy of this phototherapy [142]. The use of lipoproteins and liposomes for target delivery of PS has been also revealed to be a promising strategy [143].

The overall efficiency of PDT can be summarized in three main parameters: the photosensitizer, the appropriate source light, and the intracellular oxygen environment.

### 5.2. Photodynamic Therapy Use in Cancer

Among the uses of PDT as a therapeutic tool, the anti-cancer action of this technology is perhaps the most outstanding valence. The basis of this treatment relies on the dependence upon the uptake of the photosensitizer into the malignant tissue following administration, either systemic, topical, or intra-localized. After this step of optimal tissue accumulation, the malignant tissue can then be exposed to specific wavelengths of light for a pre-determined time for the treatment to be effective. PDT has been used since the beginning of its discovery in cancer treatment. It is no accident that the first PS used in PDT, hematoporphyrin, was directed against animal tumors [144]. Since then, the research for new compounds corresponding to the requirements of a PS and for new combining strategies to improve their inner capacity have had a deep focus in their effects in tumors with the objective to surpass some of the limitations of traditional PDT in cancer therapeutics.

Depending on the type of tumor, different PDT approaches have been successfully applied. Very recently, the first reported clinical trial was conducted to evaluate the performance of PDT in the treatment of primary breast cancer in humans [145]. In this trial, verteporfin was used as a photosensitizer at a concentration of 0.4 mg/Kg. The clinical practice involved the perforation of breast tissue by ultrasound guidance for a direct delivery of the light source into the tumor tissue. The total light dose delivered was between 20 and 40 J. After the treatment, the results revealed that about 66% of the patient’s tumors (8 in a total of 12), showed significant signs of tissue necrosis. In some cases, the tumor reduction yield was more than 10%. The authors claim that the research should be focused on the way of delivery PDT rather than trying to continuously improve the specificity of PS against a certain type of tumor, even if (like in this case) invasive measures are to be taken. Nevertheless, the most recent advances have also been focused on the latter approach. A very recent study using breast cancer cell lines and animal models, demonstrated that the use of a composite of amphiphile peptide nanomicelles loaded with indocyanine green (PAIN) used in a combinatory photodynamic/sonodynamic therapy resulted in the elimination of cancer cells both in vitro and in vivo [146]. More specifically, the authors evaluated the capability of cell elimination of PAIN after the action of photo- and sono-irradiation through the measurement of ROS cellular content. A remarkably higher cytotoxicity and consequent lower viability was found in the group treated with PAIN followed by photodynamics. To further validate its availability for breast cancer treatment, PAIN was administered into the MDA MB-231 tumor-xenografted nude mice for assessing the biodistribution in vivo. PAIN exhibited excellent body distribution and tumor targeting capability in vivo. The authors concluded then that this composite associated with a combinatorial dynamic therapy proved to be a highly efficient pathway to target and eliminate cancer cells both in vitro and in vivo. Other strategies to further enhance the delivery of PDT drugs into breast tumors involved systems based on liposomes or the use of albumin nanoparticles [147,148].

Lung cancer has long been one of the main targets of photodynamic therapy approaches [149,150,151]. The latest reviews on this matter have described new approaches to enhance the delivery of the PS to the target tissues. Multidrug resistance has proved to be a major issue in lung cancer treatment. The improvement of this targeted delivery to the exact local of tumor cells can be a colossal anti-cancer advantage. Indeed, a very recent study, showed that PLGA-lipid hybrid nanoparticles have potential to deliver photosensitizer or chemotherapeutic drug for treating both drug-selected and metastasis-associated multiple drug resistance (MDR) lung cancer cells [152]. A photosensitizer, 5,10,15,20-Tetrakis(4-hydroxy-phenyl)-21H,23H-porphine (pTHPP) was loaded into poly(D,L-lactide-co-glycolide) (PLGA)-lipid hybrid nanoparticles. The PLGA-lipid hybrid nanoparticles were showed not only to enhance the cellular uptake of the photosensitizer but to also induce apoptosis in both sensitive and MDR lung cancer cells, after light activation. The authors concluded that the drug delivery capacity of the PLGA-lipid hybrid nanoparticles was the major factor contributing to the MDR-overcoming activity of pTHPP-PLHNPs/PDT. Furthermore, they suggest that these nanoparticles may be useful not only for PS and PDT but also for the targeted delivery of non-PDT drugs such as chemotherapeutic agents. Other ways of improving PDT for lung cancer have also been involving the enhancement of light source efficacy. In a recent clinical trial, a new laser probe was tested. A composite-type optical fiberscope was used with endobronchial photodynamic therapy against peripheral type lung cancers [153]. The approach involved the use of talaporfin as PS, administered 4–6 h prior to the laser irradiation for PDT. This probe allowed not only the accurate irradiation of the lesion but also a simultaneous visualization of it. The results showed a complete response to the treatment after one year, suggesting a high efficacy of this approach. In another study, indocyanine green, was encapsulated with erlotinib-modified chitosan nanoparticles (GECs). These GECs showed synergistic molecular targeted and photodynamic therapeutic effects in inhibition of cellular growth and induction of apoptosis in non-small-lung-cancer cells. The study demonstrated the potential of combining chemo and photodynamic therapy for the treatment of lung cancer [154].

Many other types of cancer have been the focus of PDT either as primary or complementary treatment, and the trend is similar in the majority of the new approaches. Gastric, colorectal, urogenital, bladder, and pancreatic cancer, besides previously discussed breast and lung cancers, are among the ones where PDT therapies have been deeply researched, either looking to improve the delivery of the known photosensitizers or oxygen, or improve the capability of the light source [155,156,157,158,159,160,161]. Nevertheless, the search for new photosensitizers is also an active target for the development of PDT. Several works have reported the use of new compounds as promising photosensitizers [162,163,164,165,166,167,168,169,170]. From synthesized compounds based on previous structures to entirely new compounds, the research is in continuously update. Furthermore, not only lab-made compounds are being reported. The search for natural options is a growing area for photodynamic photosensitizers, and in fact natural compounds play a significant role in the development of photodynamic drugs. Although the examples given below are very recent, the search for natural compounds as photosensitizers for PDT has been active for some years [130,171]. Some examples are hypericin, hypocrellin, riboflavin, and curcumin.

### 5.3. The Use in Eye Degenerative Diseases

Ophthalmic PDT was developed in the 1990s as a treatment for age-related macular degeneration (AMD). The standard of care for several disorders of the retina is retinal photocoagulation. The photophysical properties of endogenous chromophores are used to cause photocoagulation of retinal tissues to treat proliferative diabetic retinopathy, neovascular form of AMD or macular edema. In the macula, the critical pigments that absorb light and their respective peak absorption spectra are xanthophylls (420–500 nm) within the neurosensory retina, melanin (400–1000 nm) within the RPE cells and choroidal melanocytes, and hemoglobin (450–550 nm) within red blood cells, contained within the retinal and choroidal vessels or within areas of extravasated blood [172].

Using visible wavelengths, both green (495–570 nm) and yellow (570–590 nm), or near infrared laser sources, a therapeutic photocoagulation of unwanted blood vessels is induced in the retina or by retinal photocoagulation prevent retinal detachment [173]. The depth of penetration is dependent on the incident wavelength, e.g., optical radiation from argon lasers (457–524 nm) is primarily absorbed by hemoglobin and oxyhemoglobin in retinal blood vessels, and melanin in the RPE while that from krypton red (around 650 nm) and diode lasers (790–830 nm) is absorbed by the RPE, as well as choroidal melanocytes and blood [172].

Considering external agents, human clinical trials were conducted with benzoporphyrin derivative (verteporfin), tin ethyl etiopurpurin (purlytin), and lutetium texaphyrin (Lu-tex). Verteporfin emerged as the optimal agent because of its absorption spectrum, lipophilic characteristics and short serum half-life (minimizing the duration of photosensitivity).

In PDT treatment with verteporfin activation by laser is typically performed 15–20 min after the intravenous injection of the dye [174]. A beam of red laser light (689 nm diode laser) is applied to the retina via a slit-lamp delivery system, irradiating a spot chosen to exceed the dimension of the neovascularization minimally, with light intensity of 600 mW/cm^2^, for 83 s, resulting in a total radiant exposure of 50 J/cm^2^. Closure of abnormal (leaking) blood vessels occurs for approximately 6–12 weeks in most patients. Inactive verteporfin showed a dose-dependent increase in toxicity for primary human scleral fibroblasts at 1 µg/mL and above [175]. This may constitute a drawback of these type of photosensitizers. As so, natural photosensitizers may arise as a solution.

### 5.4. Anthocyanins and Their Derivatives, A Potential PDT Ally?

Despite the fact that numerous types of photosensitizers have been discovered and developed over the years, only a small number of these compounds have been clinically approved being predominantly influenced by the tetrapyrrole structure. The reason for such is that modern PDT has its roots in porphyrin-derived agents, and a large number of preclinical and clinical experiences have been accumulated predominately on the basis of photofrin-mediated PDT. This fact may be the main reason for the continuous research on the improvement on the delivery of the pre-existing compounds. However, the search for new options should be considered.

Shi et al. have shown the potential use of traditional plants extracts for PDT in the treatment of head and neck squamous cell carcinoma [163]. From the initial 289 plant extracts, 13 showed high fluorescence properties and were screened for their photo-physical characteristics. From these, *Acanthopanacis Cortex* extract was showed to have potential photosensitizer properties, as revealed by the cell viability and intracellular distribution assays performed in KB, Hep-2 cells. The authors concluded that the extract of *Acanthopanacis Cortex* irradiated at 625 nm could enhance PDT by inducing ROS and apoptotic pathways in the tested cell lines. This reinforces the perspective about the potential use of natural compounds.

In therapeutic PDT, the photodynamic sensitizers should absorb between 600 and 800 nm (red light). Although, and similar to tissue chromophores (amino acids, nucleotides, heme, melanin, etc.), anthocyanins at acidic pH absorb light at λ < 600 nm (UV-visible), which may at first exclude their applications for therapeutic PDT.

Besides, at plasmatic pH, resonance-stabilized quinonoid anions (color purple blue of anthocyanins) are formed from further deprotonation of the quinoidal species. So, there is a shift towards red light.

In addition, the mechanism of blue color development in living flower petals involves anthocyanin-metal complexes which may result in a bathochromic shift towards the red region [176].

The photophysical and chemical properties of anthocyanins confer to this class of compounds the suitable features to be used as a photosensitizer in dye-sensitized solar cell (DSSC) [177]. In a study by Chien et al., anthocyanins were incorporated in solar systems and showed to improve their efficiency. These newly dye-sensitized solar cells boosted the system capability by almost 2% at a concentration of 3 mM of red cabbage anthocyanins and at pH 8.0, revealing a crucial importance of the anionic quinoidal bases for this phenomenon [178]. In another very recent study, the sensitization effect of anthocyanins of *Plumeria Rubia* and chlorophyll dyes on optical and photovoltaic properties of zinc oxide-based dye-sensitized solar cells (DSSC) was evaluated. The authors found that anthocyanins formed a better bound with zinc oxide and the anthocyanin-DSSC showed a higher performance compared to that of chlorophyll [179]. Dimethylamino-pyranoanthocyanins have also been proposed for the use in this type of solar technology [180].

Both properties may confer this class of compound, at least part, of the requirements for PDT.

Besides strong absorption in the red visible spectrum (600–800 nm) and a high extinction coefficient (50.000–100.000 M^−1^·cm^−1^), to prevent the need of using great amounts of PS, the photosensitizing agent suitable for PDT must be available in a pure form, and it should be easy to obtain and have a high singlet oxygen quantum yield [181].

The production of an oxygen singlet by triplet excited state of anthocyanins derivatives (pyranoanthocyanins) was recently reported [181], and the fluorescence properties of purple-fleshed sweet potato recently established [17].

Besides that, anthocyanins are fully established as potent bioactive natural compounds against a myriad of pathologies, including cancer as referred above. Altogether, these features certainly make anthocyanins a potential candidate for PDT use, as both photosensitizer and co-adjuvant for PDT enhancement. In fact, a recent study exploring the potential of cyanidin-3-*O*-glucoside as a photosensitizer for antimicrobial treatment revealed an efficient activity of this anthocyanin upon activation by green light laser [182]. In another very recent study, the modulatory effects of *Cornus mas* anthocyanin-rich extract in 5,10,15,20-terat-suphonato-phenyl-porphyrin (TSSP) based PDT was evaluated in animal models [183]. The authors found that the apoptotic and necrotic effects of the PDT treatment were enhanced by *Cornus mas* association before PDT.

As well-established antioxidants, anthocyanins may have the potential to interact with the oxygen singlet through quenching phenomena, which may be a problem for the efficiency of PDT. However, depending on the concentration of antioxidants, the hormesis effect may promote a pro-oxidative behavior. A recent study showed that several natural and synthetic antioxidants have the potential to enhance the efficacy of PDT, probably due to the quenching action driving the mechanism of PDT towards free radical rather than ^1^O_2_ [184]. Anthocyanins are reported as singlet oxygen quenchers. Cyanidin-3-O-glucoside, cyanidin-3-O-rutinoside, cyanidin-3-O-galactoside, malvidin-3-O-glucoside, and malvidin-3,5-O-diglucoside, all showed capacity to function as catalytic quenchers of ^1^O_2_ [185]. Furthermore, the stability of mono and diglucoside anthocyanins was evaluated under the presence of photochemically produced oxygen singlet. The degradation of the different anthocyanins suggested a quenching capacity to ^1^O_2_ [186]. This ability may direct anthocyanins to type I PDT, in which anthocyanins may act in their triplet state directly in biomolecules.

Although a lot more research is obviously necessary, it becomes clear that anthocyanins and their derivatives may represent an interesting new class of compounds for PDT treatment, either as primary photosensitizers or co-adjuvants.

## 6. The Role of Bioavailability for Anthocyanins Photoprotective Properties

In order to exert their biological effects, natural compounds must reach their target tissues. From a therapeutic point a view, the bioavailability of such bioactives is a crucial determinant for their expected effects [187]. In this way, the efficiency of techniques such as PDT will highly depend on the capacity of the compounds to cross the different cell barriers, either at gastrointestinal or skin level, depending on the administration route of the photosensitizer. In the case of anthocyanins this is of particular interest, due to their previous discussed physical and chemical characteristics. Anthocyanin’s bioavailability is normally considered rather low; however, today it is widely known that many factors underestimate the real bioavailability of such compounds [187]. Recent evidence suggests that these compounds can be absorbed at the gastrointestinal level and detected in the systemic circulation [120,187,188,189]. Together with their metabolites, these compounds can be detected after a few minutes after ingestion. Not only their kinetics but also their mechanisms of absorption are widely known. Bilitranslocase and glucose transporters at the gastric level and glucose transporters and intestinal level have been reported as important mechanisms of anthocyanins transport across such barriers [4,119,190,191,192]. Furthermore, the structural influence on their transport efficiency has been widely reported, and very recently it was shown that complex anthocyanins from purple-fleshed sweet potato were more resistant to digestion processes and were able to cross both gastric and intestinal cell barriers with a comparable transport to the monoglucoside anthocyanins [5]. Although their metabolization has a significant extent, this may be advantageous to these compounds has their metabolites can also exert biological effects at several degrees and targets [193].

Regarding the skin application of anthocyanins, different factors must be taken into consideration to ensure the effectiveness of their biological activities. Not only the properties of the compounds, but also the properties of the formulation itself in which they are incorporated, determine the capacity to overcome the stratum corneum (SC) and reach the epidermal and dermal skin layers. Different systems, such as ultradeformable liposome and noisome-based gels have been reported for their capacity to enhance the skin penetration, stability, and delivery of anthocyanins [84]. More recently, the release and skin permeation profiles of anthocyanins from a lipophilic delivery system were extensively studied in both porcine skin model and in human volunteers [194]. Two distinct lipstick formulations, one containing elderberry (mainly composed by simple glycosylated anthocyanins) and red radish (predominantly composed by larger anthocyanins exhibiting complex patterns of glycosylation and acylation) were tested to understand how the different structural patterns of anthocyanins might influence the skin permeation capacity. As expected, the smaller hydrophilic elderberry anthocyanins displayed higher permeability coefficient Kp (cm/h) and steady state fluxes Jss (μg cm^2^/h) across the SC following lipstick application either in human volunteers or on porcine ear skin. However, the compounds from red radish were also identified at depths relevant for overcoming the SC, which indicates that the molecular weight was not a limitation for their diffusion in the skin. Overall, both HPLC and ATR-FTIR analyses allowed the identification and quantification of anthocyanins within the samples from the volunteers, demonstrating the penetration of the compounds as well as their lateral diffusion within the skin. Interestingly, hypodermal delivery has been reported for delivery of a proanthocyanidin-rich extract from grape seeds, a strategy to reach the epidermal and dermal skin layers, overcoming the issue of SC barrier crossing [195]. The quantification of anthocyanins within the epidermis and dermis is still a relatively unexplored topic of research, but it is of major importance considering the need to determine the required concentrations to observe the beneficial properties of anthocyanins in vivo, therefore this issue should be extensively explored in future works as it would provide very useful insights regarding the topical application of these molecules. Altogether, the actual knowledge about anthocyanins bioavailability reinforces the potential use of these natural compounds’ novel photosensitizers for PDT and other skin applications.

## 7. Conclusions

**Figure 6:**
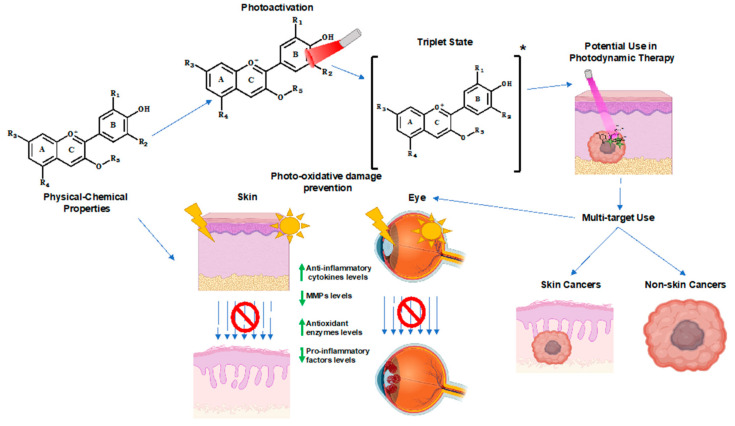
The potential applications of the photoprotective properties of anthocyanins and their related derivatives in biological systems. R_1_ = H, OH or OCH_3_; R_2_ = H, OH or OCH_3_; R_3_ = OH or sugar moiety; R_4_ = OH or sugar moiety; R_5_ = sugar moiety. The sugar moiety of anthocyanins can be composed by different attached molecules. [Anthocyanin]* denotes the triplet state.

Anthocyanins are generally associated with their antioxidant effect; although, anthocyanins are more than that, and they are quite versatile pigments with unrevealed properties (Figure 6). These pigments have been a challenge not only for chemists but also for biologists, especially due to their equilibrium forms at different pHs. This feature is clearly amazing since the observed biological effect at each pH is a net of the individual effect of each molar fraction. This scenario is even more complex when the diversity of natural sources is taken in consideration and with them an extraordinary huge number of related structures. Due to their ability to absorb light in the UV and blue light region and to revert the pharmacological pathways associated with UV prolonged exposure, anthocyanins possess the ability to protect skin and eye cells against light damage.

Anthocyanin’s dual oxidative effect may be explored towards phototherapy. In fact, due to the reported ability to form quinones and quench singlet oxygen, these pigments may be used in the PDT type I mechanism.

One of the main properties of anthocyanins is their reactivity which is dramatically reduced with the formation of a 4th ring through chemical pathways identified in anthocyanin-rich foodstuffs, such as red wine. These derivative pigments, pyranoanthocyanins, present a wide spectrum of colors ranging from yellow orange to blue, extending the range of absorption in the visible range from 400 to 500 (precursor pigments) to 600–800 nm. In addition, the ability to produce singlet oxygen, not reported for anthocyanins, may include these anthocyanin-derivative pigments as PS in type II mechanism.

In conclusion, anthocyanins constitute a natural source of unexplored bioactives towards photoprotection.

## Figures and Tables

**Table 1:** Summary of the photoprotective effects of anthocyanins in different models.

Sources	Anthocyanin Components	Study Models	Biological Effects	Reference
—	Delphinidin-3-*O*-glucoside	HaCat human keratinocytes; SKH1-hairless mouse skin; Human dermal fibroblasts; JB6 P+ mouse keratinocytes	↓ UVB-mediated oxidative stress and DNA damage; ↓ UVB-induced MMP-1 expression; ↓ UVB-induced COX-2 expression (via MAPKK4 and PI-3K targeting) and PGE2 production	[78,79,80]
—	Cyanidin-3-*O*-glucoside	JB6 P+ mouse keratinocytes	↓ UVB-induced COX-2 expression (via MKK4, MEK1, and Raf-1 targeting) and PGE2 production	[81]
—	Cyanidin-3-*O*-glucoside	Human keratinocytes	↓ UVB-induced cellular morphology change and toxicity ↓ UVB-induced ROS generation ↓ UVB-induced COX-2 expression	[82]
Topical formulation with blueberry extract (*Vaccinium myrtillus*)	n.s.	HaCat human keratinocytes; Zebrafish; Human skin explants	↑ penetration of the stratum corneum (liposomal formulation) ↓ UVC and UVA-mediated cytotoxicity ↑ wound repair	[84]
Bog blueberry extract (*Vaccinium uliginosum* L.)	Cyanidin, Petunidin, Malvidin and Delphinidin-3-*O*-glucosides; Delphinidin-3-*O*-arabinose	Human dermal fibroblasts (HFF-1)	↓ UVB-induced MMP-1, MMP-8, and MMP-13; ↓ UVB-induced production of pro-inflammatory cytokines TNF- α, IL-8, IL-6, and IL-1β	[85]
Purple-fleshed sweet potato extract	Cyanidin and Peonidin-3-(*p*-hydroxybenzoyl)-*O*-sophoroside-5-*O*-glucosides; Cyanidin and Peonidin 3-(caffeoyl)-*O*-sophoroside-5-*O*-glucosides; Cyanidin and Peonidin 3-(feruloyl)-*O*-sophoroside-5-*O*-glucosides	BALB/c-nu mouse skin	↓ UVB-induced oxidative stress Prevention of UVB-induced water loss, collagen degradation, epidermal hyperplasia and wrinkle formation; ↓ UVB-induced production and pro-inflammatory cytokines TNF-α and IL-6;	[86]
Topical formulation with strawberry extract enriched with coenzyme Q10	Pelargonidin and Cyanidin-3-*O*-glucosides; Pelargonidin-3-(malonyl)-*O*-glucoside; Pelargonidin-3-*O*-rutinoside	Human dermal fibroblasts (HuDe)	↓ UVA-mediated cytotoxicity	[92]
Seed coat of black soya bean extract	Cyanidin, Peonidin and Delphinidin-3-*O*-glucoside	—	↓ Human and mushroom tyrosinase	[95]
Rose of Sharon (*Hibiscus syriacus* L.)	Cyanidin-3-*O*-glucoside, Cyanidin-3-*O*-galactoside, Cyanidin-3,5-*O*-diglucoside	Mouse melanocytes (B16F10); Zebrafish	↓ Melanogenesis by activation of ERK signaling pathway	[96]
Bilberry extract	Delphinidin 3-galactoside delphinidin 3-glucoside, cyanidin 3-galactoside, delphinidin 3-arabinoside, cyanidin 3-glucoside, cyanidin 3-arabinoside, petunidin 3-glucoside, malvidin 3-glucoside malvidin 3-arabinoside	Human adult RPE cells (ARPE-19)	↓ Photooxidation of pyridinium bisretinoid A2E	[103]
Bilberry (*Vaccinium myrtillus*)	-	Human adult RPE cells (ARPE-19)	↑ Upregulate the oxidative stress defense enzymes HO-1 and GST-pi	[104]
Maqui berry extract (*Aristotelia chilensis*)	Delphinidin 3,5-O-diglucoside and delphinidin 3-O-sambubioside-5-O-glucoside	Murine photoreceptor cells (661W)	↓ Light-induced photoreceptor degeneration by inhibiting phosphorylation of p38	[105]
Blueberry extract	Malvidin, malvidin-3-glucoside and malvidin-3-galactoside	human retinal capillary endothelial cells (HRCECs)	↑ SOD and catalase ↓ Inhibition of ICAM-1 and NF-κβ	[106]
Blueberry anthocyanins bilberries (*Vaccinium myrtillus*)		Rats	↓ Diabetes-induced retinal abnormalities through Nrf2/HO-1 signaling, ↓ Decreased retinal vascular endothelial growth factor (VEGF) expression ↑ Degradation of zonula occludens-1, occludin and claudin-5	[107,108]
Black soybean seeds		Rat retinal neurons	↓ damage induced by N-methyl-N-nitrosourea	[109]
